# The physiological role of drug transporters

**DOI:** 10.1007/s13238-015-0148-2

**Published:** 2015-03-24

**Authors:** Yu Liang, Siqi Li, Ligong Chen

**Affiliations:** 1 Department of Pharmacology and Pharmaceutical Sciences, School of Medicine, Tsinghua University, Beijing, 100084 China; 2 Collaborative Innovation Center for Biotherapy, State Key Laboratory of Biotherapy and Cancer Center, West China Hospital, West China Medical School, Sichuan University, Chengdu, 610041 China

**Keywords:** transporter, physiological role, therapeutic implication

## Abstract

Transporters comprise the largest family of membrane proteins in human organism, including members of solute carrier transporter and ATP-binding cassette transporter families. They play pivotal roles in the absorption, distribution and excretion of xenobiotic and endogenous molecules. Transporters are widely expressed in various human tissues and are routinely evaluated during the process of drug development and approval. Over the past decade, increasing evidence shows that drug transporters are important in both normal physiology and disease. Currently, transporters are utilized as therapeutic targets to treat numerous diseases such as diabetes, major depression, hypertension and constipation. Despite the steady growth of the field of transporter biology, more than half of the members in transporter superfamily have little information available about their endogenous substrate(s) or physiological functions. This review outlines current research methods in transporter studies, and summarizes the drug-transporter interactions including drug-drug and drug-endogenous substrate interactions. In the end, we also discuss the therapeutic perspective of transporters based on their physiological and pathophysiological roles.

## Introduction

Movement of many drugs and endogenous molecules across the cell membrane is governed by protein transporters. In general, membrane transporters are divided into two major superfamilies—the ATP binding cassette (ABC) family and the solute carrier (SLC) family. To date, more than 400 transporters have been annotated in the human genome. The ABC transporters are primary active transporters that utilize the energy from ATP hydrolysis to transport substrates across the membrane. SLCs can be either facilitative transporters, which transport their substrates down the gradient across the membrane, or secondary active transporters, which transport their substrates against the gradient across the membrane by coupling a downhill transport of another substrate. Major drug transporters are listed in Table 1 together with their relevant abundance among human tissues, physiological roles, endogenous and exogenous substrates, and related pathologies.

**Table 1:** Biological profiles of major drug transporters: expression, substrates, physiology and pathology*

Gene	Protein (alias)	Tissue	Physiological roles	Related pathology**	Physiological substrate	Drug substrate
ABCB1	MDR1/P-gp/ABCB1	Ubiquitously expressed Major: intestine, brain, liver, kidney	Export of xenobiotics from cells into extracellular spaces (e.g. at the BBB) or out of the body (e.g. in the gut) and for renal and hepatic clearance	Colchicine resistance,uterine sarcoma, soft tissue sarcoma, breast cancer, inflammatory bowel disease 13, lung cancer, acute myeloid leukemia, myeloma, warfarin sensitivity, postural hypotension, cannabis dependence, vaginitis, plasmablastic lymphoma, pervasive developmental disorder, microsporidiosis, ileus, neonatal abstinence syndrome, 5-fluorouracil toxicity, paralytic ileus, engraftment syndrome, ovarian cystadenocarcinoma, acute non lymphoblastic leukemia	Steroids, lipids, bilirubin, bile acids	Digoxin, loperamide, berberine, irinotecan, doxorubicin, vinblastine, paclitaxel, fexofenadine, seliciclib
ABCB11	BSEP/ABCB11	Major: liver; others: intestine, kidney, placenta, testis, brain	Disposition of bile salts from the liver, into the bile canaliculi for export into the gut	Cholestasis, progressive familial intrahepatic 2, benign recurrent intrahepatic cholestasis 2, cholestasis, intrahepatic cholestasis, liver disease, low gamma-Gt familial intrahepatic cholestasis, intrahepatic cholestasis of pregnancy, benign recurrent intrahepatic cholestasis, lung cancer, colchicine resistance, recurrent intrahepatic cholestasis of preganancy, Abcb11-related intrahepatic cholestasis	Bile acids	Pravastatin, vinblastine
ABCC1	MRP1	Ubiquitously expressed testis, cardiomyocytes, placenta, prostate, lung, thymus and kidney, with lower expression in small intestine, colon, brain	Efflux of xenobiotic and endogenous metabolites; transport of inflammatory mediators (e.g. LTC4)	Pseudoxanthoma elasticum, Dubin-Johnson syndrome, cholestasis, lung cancer, intraocular lymphoma, microsporidiosis, colchicine resistance, cholangiolocellular carcinoma, intraocular retinoblastoma, acute myeloid leukemia	Leukotrienes, prostaglandins, 15-deoxy-D12,14 hydroxynonenal-SG, folic acid, leucovorin, GSH, GSSG, N-acetyl-Leu-Leu-norleucinal bilirubin, sphingosine 1-phosphate, glucuronide conjugates of: 17β-estradiol, bilirubin, hyodeoxycholate, dehydroepiandrosterone sulfatolithocholate, sulfatolithocholyl taurine	Adefovir, indinavir, saquinavir, ritonavir methotrexate, edatrexate, tomudex, doxorubicin, daunorubicin, epirubicin, idarubicin, etoposide, vincristine, vinblastine, paclitaxel, irinotecan, SN-38 flutamide, hydroxyflutamide, romidepsin, apicidin, fifloxacin, grepafloxacin, ciprofloxacin, berberine, pirarubicin, sodium arsenite/arsenate, potassium antimonite/antimony tartrate, citalopram, GSH conjugates of 2,4-dinitrophenyl bimane-, N-ethylmaleimide, doxorubicin, thiotepa, cyclophosphamide, melphalan, chlorambucil, ethacrynic acid, metolachlor, atrazine, sulforaphane, aflatoxin, B1, 4-nitroquinoline 1-oxide-, arsenic, glucuronide conjugates of, etoposide, NNAL, SN-38, E3040S
ABCC2	MRP2	Major: liver, kidney, intestine; Others: gallbladder, bronchi, and placenta	Terminal excretion and detoxification of endogenous and xenobiotic organic anionic compounds	Dubin–Johnson syndrome	Bilirubin, leukotriene C, S-Glutathionyl, 17β-estradiol, cholecystokinin peptide, ethinylestradiol-3-O-glucuronide, estrone 3-sulfate	Glutathione and glucuronide conjugates, methotrexate, etoposide, mitoxantrone, valsartan, olmesartan, glucuronidated SN-38
ABCC3	MRP3	Ubiquitously expressed Major: Liver and intestine	Physiological regulation of bile salt enterohepatic circulation, as well as the disposition of phytoestrogens	Dubin-Johnson syndrome, obstructive jaundice, extrahepatic cholestasis, cholangiolocellular carcinoma	Bile salts, estradiol-17β-glucuronide, leukotriene C4	Fexofenadine, methotrexate, vincristine, teniposide, etoposide, acetaminophen glucuronide
ABCC5	MRP5	Ubiquitously expressed	May be involved in cellular signaling by eliminating camp and cgmp from the cells, protective function against xenobiotics	Lymphoblastic leukemia, dysembryoplastic neuroepithelial tumor	cAmp, cGmp, folate, hyaluronan	Methotrexate, 6-, 6-thioguanine, 9-(2-phosphonyl- methoxyethyl)-adenine (PMEA), 5-fluorouracil, rosuvastatin, atorvastatin
ABCG2	BCRP/ABCG2	Intestine, liver, kidney, brain, placenta, mammary glands	Regulation of intestinal absorption, biliary and renal secretion of substrates and protection of the fetus and brain from toxins; a major role in the multidrug resistance	Breast cancer, choriocarcinoma, erythroplakia, acute lymphocytic leukemia, dysembryoplastic neuroepithelial tumor, adult acute lymphocytic leukemia, nonpapillary renal cell carcinoma, acute myeloid leukemia	Dietary flavonoids, porphyrins, estrone 3-sulfate, uric acid	Anthracyclines, daunorubicin, doxorubicin, topotecan, SN-38, irinotecan, methotrexate, imatinib, irinotecan, mitoxantrone, nucleoside analogs, prazosin, pantoprazole, statins, topotecan
SLC10A1	NTCP	Liver	Uptake of bile acids into the hepatocytes from the sinusoids	Biliary atresia, hepatoblastoma, erythropoietic protoporphyria, primary biliary cirrhosis	Bile salts, sulfo-conjugated bile acids, sulfated steroids, sulfated thyroid hormones, bromosulphthalein	Rosuvastatin, atorvastatin, pitavastatin, fluvastatin
SLC10A2	ASBT	Major: intestine, kidney, gallbladder	Bile acids absorption	Primary bile acid malabsorption	Bile acids, taurine- and glycine-conjugated bile acids	Dimeric bile acid analogues benzothiazepine derivates benzothiepene derivates and naphtol derivates
SLC22A1	OCT1	Major: liver	Uptake of cationic compounds across the sinusoidal membrane into hepatocytes; reabsorption of cationic compounds in kidney.	Chronic myeloid leukemia	Thiamine, choline, acetylcholine, agmatine, monoamine neurotransmitters	Metformin, oxaliplatin, aciclovir, ganciclovir, lamuvidine, pentamidine, furamidine, berberine, picoplatin, cis-diammine(pyridine) chloroplatin (ii), irinotecan, palitacel
SLC22A2	OCT2	Major: kidney; others: small intestine, lung, skin, placenta, brain	Uptake of organic cationic compounds for renal excretion; reabsorption of choline, dopamine etc. In the proximal tubule; involved in passage of drugs across the blood-brain barrier and distribution in neurons	Choriocarcinoma	Creatinine, bile acids, choline, acetylcholine, dopamine, norepinephrine, epinephrine, serotonin, histamine, agmatine, putrescine, cyclo (His-Pro), salsolinol	Metformin, pindolol, procainamide, ranitidine, amiloride, oxaliplatin, varenicline cisplastin, debrisoquine, proplanolol, guanidine, D-tubocurarine, pancuronium, mementine, amantadine, picoplatine, ifosfamide, cimetidine, famotidine, zalcitabine, lamuvidine, berberine
SLC22A3	OCT3	Ubiquitously expressed Major: kidney, liver, placenta, heart, and skeletal muscle	Uptake of organic cationic compounds into brain, heart and liver; involved in the distribution of cationic compounds in the brain	Not clear	Creatinine, carnitine, choline, guanidine, acetylcholine, dopamine, norepinephrine, epinephrine, serotonin, histamine, corticosterone, progesterone, testosterone, agmatine	Lidocaine, atropine, etilefrine, lamuvidine, phenoxybenzamine, prazosin, diphenylhydramine, metformin, cimetidine, ranitidine, amantadine, ketamine, memantine, phencyclidine, nicotine, clonidine, etilefrine,o-methylisoprenaline, dizocilpine, verapamil, procainamide, citalopram, desipramine, imipramine, granisetron, tropisetron, quinine, quinidine, mitoxantrone, d-amphetamine
SLC22A4	OCTN1	Ubiquitously expressed	Involved in the reabsorption of zwitterions and the secretion of cations in the proximal tubule	Rheumatoid arthritis	Ergothioneine, carnitine, acetylcholine, glycine-betaine	Pregabalin, tiotropium, ipratropium, pyrilamine, quinidine, quinine, verapamil, doxorubicin, mitoxtantrone, gabapentin, oxaliplatin, ipratropium, stachydrine, betonicine
SLC22A5	OCTN2	Ubiquitously expressed. Major: intestine, kidney	Uptake of carnitine into small intestine, kidney, adipocytes, cardiac myocytes, skeletal muscle cells, neurons, brain, lymphocytes, spermatozoa, and across the blood-retinal barrier	Crohn’s disease, primary systemic carnitine deficiency, hypoglycemia, visceral steatosis, neutral lipid storage disease, primary sclerosing cholangitis, lipid storage disease	Carnitine, choline	Etoposide, cephaloridine, ipratropium, tiotropium, mildronate, emetine, verapamil, spironolactone, pyrilamine, oxaliplatin
SLC22A6	OAT1	Major: kidney; others: choroid plexus in brain, skeletal muscle and placenta	Uptake of urate and other anionic compounds for renal excretion	Bipolar I disorder	Medium chain fatty acids, citrulline, cAmp, cGmp, prostaglandin E2, F2, urate, vanilmandelic acid, sulfated flavonoid conjugates, hydroxicinnamic acids	Adefovir, zidovudine, ciprofloxacin, cephaloridin, methotrexate, pravastatin, antibiotics, antivirals, H2 blockers, diuretics, nonsteroidal anti-inflammatory drugs, statins
SLC22A7	OAT2	Liver, Kidney	Efflux of glutamate into the sinusoids and uptake of various organic anion compounds into hepatocyte; tubular secretion of urate and organic anion compounds; possibly involved in regulation of intracellular cgmp content	Citrullinemia, citrin deficiency	Glutamate, glutarate, urate, ascorbate, orotic acid, trigonelline, hypoxanthine, GMP, GDP, GTP, cGmp, cAmp, prostaglandin E2, F2, estrogen-3-sulfate, dehydroepiandrosterone sulfate, a-ketoglutarate	Salicylate, bumetanide, erythromycin, tetracyclin, zidovudine, ranidine, 5-fluorouracil, paclitaxel, allopurinol, methothrexate, taxol
SLC22A8	OAT3	Kidney, brain, skeletal muscle, developing bone	Involved in renal secretion of urate and indoxyl sufate and anion drug metabolites; involved in removal of neurotransmitter and anion drug metabolites from brain	Not clear	cAmp, cortisol, prostaglandin E2, F2a, dehydroepiandrosterone sulfate, estrone sulfate, estradiol-17β-glucuronide, taurocholate, cholate, urate, indoxyl sulfate, flucuronide conjugates of flavonoids, vanilmandelic acid, hydroxycinnamic acid conjugates	Benzylpenicillin, tetracyclin, valacyclovir, zidovudine, adefovir, cidofovir, tenofovir, activated oseltamivir, cimetidine, famotidine, ranitidine, fexofenadine, bumetanide, furosemide, torasemide, indomethacin, salicylate, ketoprofen, ibuprofen, pravastatin, rosuvastatin, methotrexate, topotecan, quinaprilat, sitagliptin, edaravone sulfate
SLC47A1	MATE1	Major: Liver and kidney; Others: adrenal gland, testes, heart and skeletal muscle	Renal and biliary excretion of endogenous and exogenous organic cations	Unknown	Peptides and nucleosides, creatinine, guanidine, thiamine, E3S	Metformin, cephalexin, acyclovir, gancyclovir, fexofenadine, oxaliplatin
SLCO1A2	OATP1A2	Ubiquitously expressed. Major: brain, kidney, liver, intestine	Transportation of organic anionic, neutral and cationic compounds; delivery and removal of thyroid hormones	Aneurysmal bone cysts	Cholic acid, DHEAS, prostaglandin E2, taurocholate, TCDC, thyroxine triiodothyronine, bilirubin, conjugated sex steroids, linear and cyclic peptides	Erythromycin,fexofenadine, imatanib, levofloxacin and other fluoroquinolones, lopinavir, methotrexate rocuronium,rosuvastatin, pitavastatin, ouabain, saquinavir (probably other pis) deltophorin II, DPDPE, sulfobromophthalein, unaprostone, acebutolol, atenolol, atrasentan, celiprolol, sotalol,talinolol, tebipenem pivoxil
SLCO1B1	OATP1B1	Liver	Involved in drug disposition and is responsible for the hepatic uptake of drugs and endogenous compounds	Rotor syndrome, hyperbilirubinemia, rotor type, digenic	Bile acids, bilirubin, steroid hormones, thyroid hormones, steroid sulfates, glucuronid conjugates and peptides,prostaglandin E2, thyroxine (T4) and T3	Statins, repaglinide, olmesartan, enalapril, temocaprilat, valsartan, phalloidin
SLCO1B3	OATP1B3	Liver	Involved in drug disposition and is responsible for the hepatic uptake of drugs and endogenous compounds	Rotor syndrome, hyperbilirubinemia, rotor type, digenic	Bilirubin, bile acids, conjugated steroids, eicosanoids and thyroid hormones, cholecystokinin	Statins, fexofenadine, telmisartan, enalapril, erythromycin, phaloidin, valsartan, docetaxel, digoxin, paclitaxel, amanitin

* Adapted from Hagenbuch and Stieger (2013), Koepsell (2013), and Gáborík et al. (2014)

** Refer to http://www.malacards.org/ (Rappaport et al., 2013). Pathologies with Relevance Score > 1.0 are listed

The biological function of transporters has been investigated using multiple approaches. Among them, metabolomic studies have been quite valuable for substrate identification of transporters systemically, which identify the alterations of many metabolites or their substrates directly. Alternatively, the uptake of substrate candidates can be evaluated by radio-tracer or radio-free LC/MS quantification. This method was used to identify thiamine and ergothioneine as the physiological substrates of OCT1 (SLC22A1) and OCTN1 (SLC22A4) respectively (Chen et al., 2014; Gründemann et al., 2005). Clinically, genome-wide association studies (GWASs) have proved a powerful method for identifying disease susceptibility genes. GWASs indicate that genes coding for a variety of transporters are susceptibility genes for many diseases (Gottesman and Ambudkar, 2001; Stefková et al., 2004; Grisanzio et al., 2012; Suhre et al., 2011; Diabetes Genetics Initiative et al., 2007), suggesting that transporters have important physiological/pathophysiological roles in disease. The function of transporters in drug ADME (absorption, distribution, metabolism and excretion) has been intensively studied during the past two decades but their physiological roles have been much under studied. There is emerging evidence that understanding the physiological roles of transporters in normal as well as pathophysiological conditions is of great value for the development of new drugs and therapies (the International Transporter Consortium et al., 2010; Mizuno et al., 2003). In this review, we summarize recent progress in the study of physiological roles of transporters, including new methodology in transporter biology, drug-drug interaction (DDI) and drug-endogenous substrate interaction. Finally, we discuss pharmaceutical implications of transporters using the roles of transporters in metabolic diseases as examples.

## Current research trends in transporter study

With the rapid progress of biomedical technologies, methods for studying membrane transporters have expanded extensively. Generally, transporter research methods are performed using bottom-up or top-down approaches. The top-down approach utilizes computational biology or crystal structure analyses of transporter structure, which provides insight into the binding site, binding affinity prediction and the potential conformational change of the transporter upon substrate binding. Using this information, predicted agonists or antagonists for the transporter can be validated via *in vitro* transporter assays. The bottom-up approach uses discovery metabolomics, cell culture and animal models, GWAS or other genomics data as well as metabolism pathways to identify substrate candidates for specific transporters. In this section, we mainly discuss current trends in methods for studying membrane transporters.

### Structure-based computational biology

Research progress in ligand-protein interaction has been greatly aided by advances in structural biology, most notably X-ray crystallography, NMR spectroscopy and electron microscopy. In particular, computational modeling and virtual screening of small molecule libraries based on structural biology have accelerated the identification of substrates and drug candidates of transporters. Although there are only a few human SLC transporters whose atomic structures have been determined [e.g. the Rhesus glycoprotein ammonium transporter SLC42A3 (Gruswitz et al., 2010) and the glucose transporter SLC2A1 (Deng et al., 2014)], several high-resolution structures of proteins from prokaryotic and other eukaryotic organisms are available with at least 25% sequence identity with human homologues (Gao et al., 2009; Fang et al., 2009; Shaffer et al., 2009; Lu et al., 2011). Substrate prediction and virtual screening of drug candidates become possible with computational modeling and docking based on the available structural information. For example, novel ligands have been successfully screened from compound libraries for the norepinephrine transporter (NET, SLC6A2, Schlessinger et al., 2011), the GABA transporter 2 (GAT-2, SLC6A13, Schlessinger et al., 2012), and the large-neutral amino acid transporter (LAT-1, SLC7A5, Geier et al., 2013), based on the crystal structures of their prokaryotic homologues. Ligand discovery can also be accomplished by ligand-based modeling. For example, Wittwer et al. developed a quantitative structure-activity relationship (QSAR) model of ligand binding to the multidrug and toxin extrusion transporter 1 (MATE1, SLC47A1) by experimental high-throughput screening (HTS) for drug libraries. After refinement by low-throughput experiment validation, the performance of the model was improved. The refined model was then applied to the screening of compound libraries in order to find novel ligands binding to MATE1 (Wittwer et al., 2013). With the number of resolved membrane transporter structures increasing and the development of computational tools, *in silico* analysis are expected to greatly facilitate substrate or inhibitor discovery in transporter study.

### Clinical genomics

The complexity of human genetics provides a rich resource to investigate the potential roles of drug transporters in physiological and pathophysiological conditions. Human diseases linked to genetic variances, including polymorphisms, insertions and deletions, in transporter genes provide insight into their physiological roles. For example, ABCG2 was identified as a renal proximal tubular urate efflux transporter when a GWAS showed that SNPs in ABCG2 were correlated with altered serum urate levels and gout (Woodward et al., 2009). Further work confirmed that ABCG2 was a high capacity urate exporter involved in serum urate homeostasis (Nakayama et al., 2011). Many transporters have multi-specificity towards numerous substrates thus the clinical outcomes for transporters like aforementioned ABCG2 may reflect only a portion of their physiological function (Table 1). Mutations in genes that encode transporter proteins have also been associated with a variety of human diseases (as shown in Table 1 for drug transporters). For example, genetic defects in glucose transporters like GLUT1 (SLC2A1) have been associated with glucose-galactose malabsorption (Pascual et al., 2004). Mutations in anion transporter SLC26 genes are associated with congenital or early onset Mendelian diseases like chondrodysplasias, chloride diarrhea and deafness with enlargement of the vestibular aqueduct (Alper and Sharma, 2013). On the flip side, the identification of genetic variants in transporter genes with protective or beneficial effects provides substantial therapeutic targets in clinical application. An example of this is the zinc transporter ZnT8 (see discussion in the following sections in this review). Information on genetic variances in transporter genes can be obtained from a specialized database for transporter pharmacogenetics established at University of California, San Francisco (http://pharmacogenetics.ucsf.edu/). Clinical genomics also reveal involvement of transporters in bioconversion and toxicity of drugs. One of the most compelling examples of the critical role of transporters in drug toxicity stems from a GWAS encompassing 85 patients with myopathy and 90 normal control individuals (SEARCH Collaborative Group et al., 2008). This study revealed an association between the pathology of myopathy and a SNP within a non-coding region of *SLCO1B1* gene. *SLCO1B1* encodes the organic anion transporting polypeptide (OATP1B1) that has been shown to be involved in the hepatic uptake of statins. GWASs have also associated *SLCO1B1* and *SLCO1B3* variants with bilirubin levels and unconjugated hyperbilirubinemia (Johnson et al., 2009; Sanna et al., 2009), reflecting the important function of these transporters in the uptake of bilirubin in liver. Other GWASs also showed that variants in *SLCO1B1* were associated with reduced clearance of methotrexate and thus increased gastrointestinal toxicity (Treviño et al., 2009; Ramsey et al., 2012).

### *In vitro* models

Analysis of the physiological role(s) of a membrane transporter frequently begins with an *in vitro* model. Historically, the *Xenopus* oocyte has been used as a powerful expression system to study transporter activity. Due to the limited expression levels of endogenous membrane transporters, *Xenopus* oocytes show little background activity, and thus provide reliable information with high signal-to-noise ratio (Bröer, 2010). If combined with high-throughput screening technology, *Xenopus* oocytes can even serve as a drug screening system for certain transporters (Kvist et al., 2011). Equipment setup for electrophysiology for *Xenopus* oocytes and efficiency of foreign gene expression, however, are hurdles for its further application. More recently human cell lines, such as HEK-293, CHO and HepaRG cells, have been more widely used as they have metabolism characteristics that are similar to the origin tissues and it is easier to express exogenous transporters in cell lines. Moreover, vectorial transport can be studied in cultured, polarized cells like the Caco-2 and Madin-Darby canine kidney (MDCK) cells. The colon carcinoma cell line Caco-2 are especially useful for the study of intestinal transporters, since these cells exhibit morphological and functional similarity with the enterocytes lining the small intestine (Hidalgo et al., 1989). MDCK cells form a tight monolayer which separates the basolateral and apical sides similar to cells in biological barriers such as the blood-brain barrier and the placenta barrier. Such vectorial transport assays are important when investigating transport across biological barriers, from apical to basolateral side or *vice versa*.

The primary goal of transporter assays is to determine the kinetics of substrate transport. For the primary active transporter, like the ABC transporter, substrate translocation across the cell membrane is directly correlated to ATP hydrolysis. Thus ATPase assays that measure the inorganic phosphate release using colorimetric measurement can be utilized to assess active transporter kinetics (Glavinas et al., 2007). The ATPase assay is especially useful in high-throughput screening of substrate candidates as well as potent inhibitors. ATPase assays, however, are only useful for a small subset of ABC transporters in which slowly transported substrates do not stimulate detectable ATPase activity. Kinetics analysis for most types of ABC transporters requires whole-cell and vesicle transport assays, as mentioned in the following section.

For non-primary active influx transporters, the kinetics can be measured via uptake assay using intact cells. The accumulation of substrates over a certain period of time can be quantified by radio-isotope scintillation counter or LC/MS. The properties of transporters, however, must be taken into consideration when designing conditions for cellular uptake assays. For proton co-transporters, like those expressed in small intestinal epithelium, optimal transporting activity is usually observed at acidic pH condition which is close to physiological conditions in the intestine. The proton-coupled folate transporter (PCFT), encoded by SLC46A1, incorporates folate by coupling a proton co-transport down the proton gradient at pH 5.5, however, this transport activity is absent at neutral pH (Nakai et al., 2007). Peptide transporters 1 and 2 (PEPT1 and PEPT2, encoded by SLC15A1 and SLC15A2) are similar examples, which mediate absorption small peptides and peptide-like drugs from the intestine and kidney respectively with symport of a proton (Liu et al., 1995; Liang et al., 1995). There are also examples of sodium symporters whose activity relies on sodium gradient. The transport activity of such transporters, like SLC10A1, SLC10A2, SLC22A4 and SLC34A1, can be greatly suppressed when the sodium ions are replaced by choline (De Bruyn et al., 2011).

Isolated cell membranes are another reliable method to investigate transporter activity. Membranes can be prepared from insect cells, mammalian cell lines, or primary cells in which the transporter of interest is overexpressed. Insect cells like Sf9 and Sf+ are widely used because of high membrane yields and easy culturing. The major drawback of insect cells is that their cholesterol content is significantly lower than that of mammalian cells. Membrane cholesterol contributes to transporter activity, especially in the ABC transporters (ABCB11, Kis et al., 2009; ABCC2, Ito et al., 2008; ABCG2, Pal et al., 2007). Thus supplementation of cholesterol is sometimes necessary when using membranes extracted from insect cells. For efflux transporters, direct measurement of the substrate translocation can be achieved by a vesicular transport assay. Enriched, inside-out membranes are prepared using commercially available kits and incubated with the substrate and ATP or the appropriate energy source for the transporter of interest. After filtration, the vesicles containing the substrates are captured, which allows for quantification of the substrates inside vesicles via radio-isotope scintillation counter or LC/MS.

### *In vivo* models

To date, mice and rats serve as the most relevant *in vivo* model for the field of transporter biology. The recent emergence of zinc-finger nuclease (ZFN), TALEN and CRISPR/Cas-based genomic engineering provides powerful and convenient tools to generate transgenic or knockout animals for a gene of interest (Gaj et al., 2013). Beyond traditional overexpression or knockout in the whole body, tissue specific or local modification can also be achieved through floxP-Cre or Tet-on/off expression systems. Moreover, overexpression and knockdown locally in a certain tissue or at a specific developmental point can be achieved by microinjection of gene expression vectors followed by electronic transfection. Animal models are a powerful tool to study the physiological function of a transporter. Systemic overexpression or knockdown of a transporter can produce phenotypes that provide information about tissue specific functions. Some transporters are enriched in certain tissues implying that they have unique physiological functions. Thus alteration of transporter activity through genetic means may only be observed locally within a specific tissue and possibly, loss of transporter expression may be compensated by its functional orthologues or homologues. The LC/MS based metabolomics may help characterizations of local metabolite changes caused by transporter deletion or overexpression in a specific tissues or circulating system. For example, tissue extracts from liver or muscle can be used to investigate the role of a transporter in energy related metabolism; urine and serum can be used to study alterations in secreted or excreted metabolites.

## Drug-transporter interactions

### Drug-drug interactions

Due to the critical roles of transporters in the ADME of drugs, a variety of drug transporter assays are required by both FDA and EMA prior to testing of a New Chemical/Molecular Entity (NCE/NME). Potential drug-drug interactions (DDIs) may cause severe, adverse reactions in patients due to alteration in ADME by transporters. Most DDIs via transporters involve, but are not restricted to, the main transporters in drug ADME: P-gp, BCRP, OATP1B1/1B3, OAT1/3, OCT1 and OCT2 (Tweedie et al. 2013). The International Transporter Consortium has established decision trees for investigating the transporter interactions of drugs (International Transporter Consortium et al., 2010).

DDIs via a transporter can also occur in an indirect manner. The expression level and transporting activity of transporters may be altered as part of the systemic response (i.e., immune response) to exogenous or endogenous stimuli. Altered drug ADME during inflammation has long been primarily ascribed to changes of enzymes in drug metabolism. An example of this is decreased expression of P450, the main drug metabolizing enzyme in liver, following inflammation. Moreover, recent studies have also shown that the expression and activity of numerous transporters are also changed in inflammatory condition. In experimental cell and animal models, inflammatory conditions induced down regulation of MDRs, MRPs and OATPs. Consequently, the ADME of drugs which are substrates of these transporters would be broadly affected (Petrovic et al., 2007).

### Drug-endogenous substrate interactions

Although DDIs via transporters have been widely studied, the interaction between drugs and endogenous substrates has not received significant attention. The transportation and distribution of many endogenous substrates like hormones, bile acids and neurotransmitters mainly depend on the activity of transporters.

The most well-known case of drug-endogenous substrate interaction is the drug induced liver injury (DILI) due to cholestasis. The transporter BSEP plays an important role in the disposition and exportation of bile salts from liver. BSEP inhibition by drugs can cause accumulation of bile salts in liver, thus leading to cholestasis. There is a strong correlation between inhibition of the *in vitro* efflux transporter activity of BSEP and the risk of cholestatic DILI (Dawson et al., 2012). BSEP was thought to be the primary bile acid efflux transporter in normal conditions in humans (Kis et al., 2012), while MRP3 and MRP4 may play compensatory roles when BSEP is impaired (Keppler, 2011). A recent study regarding the relationship between MRP3, MRP4 and BSEP inhibition and drugs with cholestatic potential showed that inhibition of MRP4, in addition to BSEP, might be a risk factor for the development of cholestatic DILI, while the inhibition of MRP3 seemed not statistically significantly associated (Köck et al., 2014). The EMA has already included a test of BSEP transporter for potential liver toxicity.

Substrate multi-specificity of transporters and/or low amount of substrates makes altered transport of an endogenous substrate difficult to identify. Local changes in substrate homeostasis, however, may become obvious under pathological conditions. This can be due to the changes of transporter expression level under pathological conditions or because of an individual taking certain drugs. Recently it was reported that the drugs metformin and phenformin competitively inhibited hepatic thiamine uptake via OCT1, a high-capacity thiamine transporter in liver, to modulate the energy status in hepatocytes (Chen et al., 2014). OCT1 is the major hepatic uptake transporter for xenobiotics, including the most prescribed anti-diabetic drug metformin and anti-cancer drug oxilaplatin (Shu et al., 2007; Wang et al., 2002; Li et al., 2011). It has been proposed that together with drug-metabolizing enzymes, OCT1 contributes mainly to the detoxification pathway of liver (Zhang et al., 2006). But the exact physiological role of OCT1 remained speculative. Using metabolomic analysis of OCT1 overexpressed in HEK293 cells and isotopic uptake assays, thiamine was identified as one substrate, with low-affinity but high-capacity to OCT1 (Chen et al., 2014). The *Oct1* KO mice provide a model to investigate the function of Oct1 systemically. Thiamine is converted to thiamine monophosphate (TMP) and thiamine pyrophosphate (TPP) in cytosol, which serves as the essential co-enzyme for energy generation and glucose metabolism. Due to reduced thiamine uptake in liver, thiamine-related changes were observed in the serum of *Oct1* KO mice. These included reduced levels of TMP and TPP and higher levels of pyruvate and alpha-ketoglutarate, which are the substrates of pyruvate dehydrogenase (PDH) and alpha-ketoglutarate dehydrogenase (OGDH) (Chen et al., 2014), respectively. SLC19A2 (Labay et al., 1999; Diaz et al., 1999) and SLC19A3 (Rajgopal et al., 2001) are high-affinity thiamine transporters; however, OCT1 is likely the primary transporter for thiamine uptake in the liver. This is supported by the drastic reduction of thiamine uptake in *Oct1* deficient mouse primary hepatocytes (Chen et al., 2014). *Oct1* deficiency does not lead to obvious outcomes in other tissues where the uptake of thiamine is possibly controlled mainly by SLC19A2 or SLC19A3.

OCT1 has also recently been shown to be a transporter for monoamine neuro-transmitters (serotonin, dopamine, epinephrine and norepinephrine) and OCT1 might play a critical role in the uptake of serotonin in liver (Boxberger et al., 2014). The monoamine uptake transporter SERT is widely expressed in the gut where the monoamine transmitters have important physiological functions. Though liver is the major organ in which serotonin is metabolized, SERT is not expressed in liver (Ramamoorthy et al., 1993). Common OCT1-transported drugs including diphenhydramine, fluoxetine, imatinib and verapamil were shown to inhibit hepatic serotonin uptake with IC_50_ values in the low micromole range (Boxberger et al., 2014). The reduction of serotonin uptake in *Oct1* deficient primary hepatocyte was only 28% indicating that there are other transporters which contribute to the uptake of serotonin in liver (Chen et al. 2014). OCT3 and PMAT are possibly candidates as both are high-capacity serotonin transporters (Wu et al., 1998; Zhou et al., 2007); however, both have low abundance in the liver compared to OCT1. Another explanation could be that these drugs not only block OCT1 but also other transporters-mediating serotonin uptake in the liver.

The drug-endogenous substrate interaction has also been investigated in the kidney efflux transporters MATEs. Pyrimethamine, a commonly used anti-protozoal drug for both the prevention and treatment of malaria, inhibits the renal clearance of thiamine, carnitine and acylcarnitine by about 80%, 90% and 90% respectively in urine samples from patients. Nonetheless, plasma concentrations of the aforementioned compounds are not changed. Thus, alterations of thiamine and carnitine in the urine are thought to be useful as biomarkers in evaluating drug interactions via MATEs (Kato et al., 2014).

## Emerging role of transporters as therapeutic targets

Acting as the “gatekeepers” for the ADME of drugs and endogenous substrates including hormones, glucose, amino acids, inorganic ions, neurotransmitters and cellular metabolites, transporters have drawn increasing attention for drug development in recent years. Transporter-mediated therapeutics is achieved via stimulation or inhibition of the substrate transporting activity or potential cellular signaling involved. More than a dozen transporters have already been targeted in clinical therapy. These include the monoamine transporters for treatment of mental disorders like depression and schizophrenia, SGLT2 for treatment of diabetes and the MDR and MRP transporters in cancer therapy. However, much remains to be uncovered as the physiological functions of many transporters are still largely unknown. The physiological roles of transporters are generally consistent with their distribution in the body, i.e. the tissue specific expression. For example, OCT1 is mainly expressed in the central vein of liver where its physiological function is to uptake thiamine into hepatocytes (Chen et al., 2014). Most of the known functions of transporters involve inward and outward transportation of substrate across the plasma membrane. There are also transporters, however, which localize in the nuclear envelope and membranes of other organelles like the mitochondria, the lysosome, the endoplasmic reticulum and the Golgi apparatus. Investigating the physiological function of transporters is important not only for understanding the nature of substrate transportation but also for the seeking of new therapeutic targets. In this section we discuss three transporters that are potentially involved in the pathology of diabetes as examples of how a transporter’s physiological function has therapeutic implications.

### The thiamine transporter OCT1

OCT1 controls the liver uptake of thiamine whose derivative TPP serves as the essential cofactor in energy generation. The loss of *Oct1* in mice results in a 70% reduction in thiamine uptake in liver and significantly reduced fat accumulation in both lean and obese mice. This indicates that OCT1 may be a promising therapeutic target in the treatment of non-alcoholic fatty liver disease for which there are few effective therapies (Halegoua-De Marzio and Fenkel, 2014). Metformin, the most prescribed anti-diabetes drug, inhibits OCT1-mediated thiamine uptake, which corresponds well to the physiological role of OCT1 (Chen et al., 2014). This finding not only uncovered part of the pharmacological effect of metformin but also provides new therapeutic insight in the treatment of diabetes. An intriguing possibility is that by regulating the uptake of thiamine by hepatocytes, it is possible to alter the energy status in the liver and thus regulate the homeostasis of gluconeogenesis and glycolysis in diabetic patients.

### The citrate transporter SLC13A5

**Figure 1:**
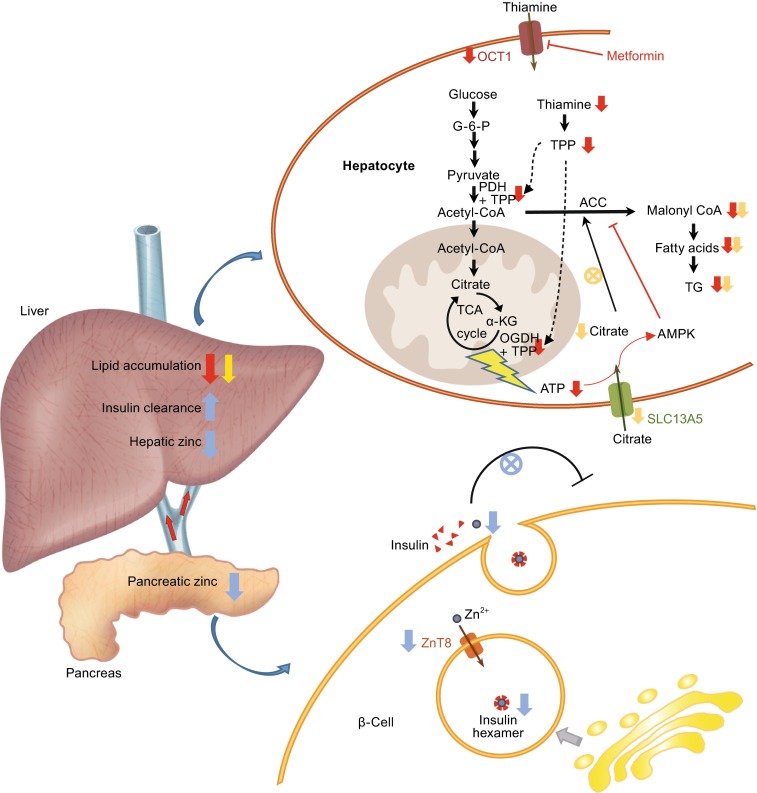
**Cartoon illustration of the physiological roles and therapeutic implications of OCT1, SLC13A5 and ZnT8 in glucose metabolism, lipogenesis and insulin pathway**. Inhibition of OCT1 (e.g., metformin treatment) results in lowered hepatic thiamine uptake, and consequently reduced TPP levels. The reduced ATP production, due to the decreased PDH and OGDH activity, triggers AMPK activation. The inhibition of SLC13A5 leads to lowered cytosol citrate contents, which attenuate the activating effect of citrate in malonyl-CoA synthesis. Both OCT1 and SLC13A5 inhibition reduce TG synthesis in the liver. Inhibition of ZnT8 causes reduced zinc release from the β-cell, and thus attenuates the autocrine effect of zinc in glucose induced insulin production; the hepatic zinc content is also reduced, with concomitant increased insulin clearance. The effects of OCT1, SLC13A5 and ZnT8 inhibition are shown with red, yellow and blue arrows respectively

In *Drosophila*, loss of INDY (I’m Not Dead Yet), the homologue of mammal SLC13A5, confers extended lifespan in a caloric restriction-like manner (Rogina et al., 2000) with reduced whole body fat stores and expression of insulin like proteins (Wang et al., 2009). SLC13A5 mediates the uptake of citrate into cytosol across the plasma membrane with high specificity. Analyses of the structure of bacterial Indy reveal a conserved substrate binding region across a wide range of species (Mancusso et al., 2012). As one of the main intermediates in the TCA cycle, citrate also regulates fatty acid synthesis and β-oxidation (Fig. 1). Hence, SLC13A5 may serve as a potential therapeutic target in the treatment of metabolic disorders including non-alcoholic fatty liver disease, obesity and diabetes. Consistent with this, loss of *Slc13a5* in mice results in reduction in the liver uptake of citrate from circulation accompanied by an increase in mitochondrial biogenesis, lipid oxidation, energy expenditure and decrease in hepatic *de novo* lipogenesis. The loss of *Slc13a5* also protects mice from high-fat diet induced obesity and insulin resistance (Birkenfeld et al., 2011). Currently, there is no available drug that specifically modulates SLC13A5 activity. Some compounds have been reported as selective inhibitors of the plasma membrane citrate transporter for both mammalian SLC13A5 and the *Drosophila* INDY, leaving mitochondria citrate transporter activity unchanged (Sun et al., 2010). A recent study reported the crystal structure of VcINDY, a homologue of SLC13A5 in *Vibrio cholera* at 3.20 Å. This provides the structural basis for understanding the substrate binding properties as well as conformational change upon substrate binding (Mulligan et al., 2014). Together with computational biology, the development of drugs targeting the SLC13A5 could be accelerated for potential therapeutics of carbohydrate and lipid metabolism disorders like diabetes, non-alcoholic fatty liver and obesity.

### Zinc transporter ZnT8

Zinc transporters are reported to be involved in the pathophysiology of cancer, cardiovascular diseases and Alzheimer’s disease making them possible therapeutic targets for treatment of these diseases (Prasad et al., 2009; Little et al., 2010; Watt et al., 2011). Normal cellular function requires tightly controlled zinc homeostasis through coordinated actions of zinc transporters and metallothioneins. Emerging evidences suggest that zinc transporters are also involved in that pathology of diabetes mellitus. Zinc plays key roles in the synthesis, storage and secretion of insulin in response to elevated glucose concentrations (Mocchegiani et al., 2008; Wijesekara et al., 2009). Of particular relevance to diabetes is the most well studied zinc transporter ZnT8 (SLC30A8). ZnT8 is responsible for the packaging and storage of insulin as Zn^2+^-insulin hexamers in the secretory granules of the pancreas, which releases the physiologically active form of insulin in the serum driven by a pH change (Xu et al., 2012b). The relationship between ZnT8 and diabetes risk has been verified in cultured cells as well as in mice. In a pancreatic cell line INS-1, ZnT8 overexpression showed enhanced glucose-stimulated insulin secretion (Chimienti et al., 2006) while down regulation of this transporter leads to reduced insulin production in response to a hyperglycemic stimulus (Fu et al., 2009). Knockout mice with global deficiency of ZnT8 showed normal glucose homeostasis and insulin production under normal conditions (Lemaire et al., 2009). Glucose tolerance and glucose-induced insulin secretion are impaired when challenged with a high-fat diet and these mice further develop diabetic obesity and insulin resistance (Lemaire et al., 2009; Nicolson et al., 2009). Mutant mice with a targeted beta cell ZnT8 deficiency display glucose-intolerance, reduced beta cell zinc accumulation and increased proinsulin levels when fed with high-fat diet, indicating the deficiency in insulin processing and secretion. These mice, however, do not develop obesity (Wijesekara et al., 2010). Genetic studies in humans also support a correlation between ZnT8 and diabetes risk. Several GWASs revealed genetic association between ZnT8 and diabetes onset (Sladek et al., 2007; Xu et al., 2012a; Diabetes Genetics Initiative et al., 2007). The most compelling evidence comes from a common SNP rs13266634 (c.973T>A, p.Trp325Arg) in Europeans which increased the risk of developing type II diabetes with concomitant impaired conversion of proinsulin to insulin (Sladek et al., 2007 and Kirchhoff et al., 2008). Cellular experiments show that the Arg325 ZnT8 variant exhibits lower apparent Zn^2+^ transport activity than the Trp325 ZnT8 (Nicolson et al., 2009). Collectively these studies indicated that reduced Zn transport activity by ZnT8 is connected to the diabetes risk. Thus treatments that increase ZnT8 transport activity might be a novel therapeutic route for treatment of diabetes.

The exact relationship between ZnT8 activity and diabetes risk, however, remains controversial. Zn ions have several effects on the regulation of insulin production and clearance. Co-secretion of Zn ions with insulin from beta cells inhibits insulin production in an autocrine and paracrine fashion through the K-ATP channel (Bancila et al., 2005; Prost et al., 2004). Beta cell-secreted Zn ions also regulate hepatic clearance of insulin. A recent study showed that beta-cell specific deficiency of ZnT8 results in low peripheral insulin level but hypersecretion of insulin from pancreas (Tamaki et al., 2013). Further, ZnT8 deficient mice and humans with the Trp325Arg ZnT8 variant exhibit increased insulin clearance. This in turn stimulates the secretion of insulin by pancreas and increases susceptibility to developing type II diabetes (Tamaki et al., 2013).

Intriguingly, a recent GWAS reported that 12, rare truncating mutations in ZnT8 were surprisingly protective against developing diabetes and, collectively, explained a 65% reduction in diabetes risk (Flannick et al., 2014). Although their study was originally performed in the European population with SNP rs13266634 genetic background, it was shown that this haplotypic background did not influence the conclusion (Flannick et al., 2014). Though the exact mechanism remains elusive, the size of the GWAS data pool (~150,000 individuals across five ancestry groups) legitimizes the finding. A recent review of the paper pointed to the unclear association between increased insulin receptor binding and liver insulin action with lower concentration of Zn ions (Pearson, 2014). Clearly, the roles of ZnT8 in Zn homeostasis and diabetes are more complicated than expected. On the one hand, ZnT8 is essential for insulin processing and secretion in response to glucose challenge thus total loss of its function causes glucose-intolerance. On the other, secreted Zinc ions into liver portal vein indeed regulate the gluconeogenesis and insulin pathway in hepatocytes. The GWAS by Flannick et al. reveals an alternate view of ZnT8 and diabetes risk. Different from OCT1 and SLC13A5, which show local control of carbohydrate and lipid metabolism in the hepatocytes, ZnT8 shows a systemic regulation of insulin pathway in the pancreas and liver (Fig. 1). Thus, drug candidates that act on ZnT8 would be expected to be potent novel anti-diabetes therapeutics.

## Summary and future perspectives

Transporter biology is a rapidly changing field in pharmaceutical research. Identifying the function of transporters in physiological and pathological conditions is garnering significant attention for drug development. Many drugs, nutrients and metabolites are moved by transporters not only through the plasma membrane but also among subcellular organelles like vesicles, ER, lysosome and mitochondria. Transporters remain one of the most underestimated fields for drug targets. Within the hundreds of annotated transporters in human genome, more than half are orphan transporters with unknown substrates or function. The nature of these transporters, including their expression and localization in organs and tissues, subcellular localization, endogenous substrates, largely remains elusive. As discussed in this review, transporters can be of significant therapeutic potential in the treatment of various diseases. A comprehensive dissection of the physiological roles of the transporter could be a key step to excavate this “gold mine”. Our growing knowledge of transporter biology will greatly accelerate the discovery of novel drug targets and development of first-in-class therapies.
